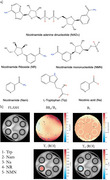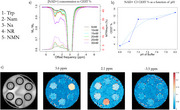# Adenosine‐Linked CEST MRI Signatures of NAD⁺ Biosynthesis Precursors for Neurodegenerative Disease Detection

**DOI:** 10.1002/alz70861_109034

**Published:** 2025-12-23

**Authors:** Festus Mathuen Slade, Wilfred W. Lam, Joanna F Collingwood, Greg Stanisz

**Affiliations:** ^1^ Physical Sciences, Sunnybrook Research Institute, Toronto, ON Canada; ^2^ University of Wawrick, Department of Chemistry, Coventry, Warwickshire UK; ^3^ Medical Biophysics, University of Toronto, Toronto, ON Canada; ^4^ University of Warwick, School of Engineering, Coventry, Warwickshire UK; ^5^ Warwick Centre for Doctoral Training in Analytical Science, Coventry, Warwickshire UK

## Abstract

**Background:**

Disruptions in NAD⁺ and purine metabolic pathways are implicated in neurodegenerative diseases such as Alzheimer’s. Chemical Exchange Saturation Transfer (CEST) MRI offers a non‐invasive modality to track these metabolic signatures in vivo. Identifying CEST properties of NAD⁺ and its precursors may enhance diagnostic sensitivity.

**Method:**

In vitro CEST MRI experiments were conducted using 3 mM agarose phantoms containing 30 mM of NAD⁺, NMN, NR, Na, Nam, or Trp in phosphate buffer across pH 5.5–8.0. Scans at 7T and 9.4T (Bruker BioSpin) were performed at 37 ± 0.2°C. Z‐spectra were acquired using saturation transfer‐prepared FLASH (7T) and RARE (9.4T) sequences with B₁ = 0.3–1.5 µT and Δω = ±7ppm. B₀ inhomogeneity was corrected using WASSR. T₁/T₂ were measured and Z‐spectra were fitted with a three‐pool Bloch–McConnell model.

**Result:**

NAD⁺ exhibited a prominent CEST peak at 2.03 ppm attributable to the adenine amine group, with up to 15% contrast and positive pH dependence at 2.98 ppm (amide). This 2.03 ppm signal—arising from the adenine ring common to NAD⁺, ATP, and adenosine—suggests potential for broader application in imaging purine metabolism. NMN also showed distinct peaks at 0.7, 2.92, and 4.42 ppm. Tryptophan and NR demonstrated pH‐sensitive exchange, while most metabolites followed base‐catalyzed exchange mechanisms. Peak separation and spectral clarity were enhanced at 9.4T, supporting further exploration of high‐field imaging.

**Conclusion:**

The 2.03 ppm adenine‐associated CEST peak from NAD⁺ represents a promising biomarker for imaging adenosine‐linked metabolic pathways, including ATP and related purines. This in vitro characterisation highlights the feasibility of targeting this signal to non‐invasively probe purine metabolism in neurodegeneration. These findings lay the groundwork for future in vivo studies investigating ATP, cAMP, and NAD⁺ dynamics via CEST MRI.